# Symptoms of posttraumatic stress disorder among targets of school bullying

**DOI:** 10.1186/s13034-019-0304-1

**Published:** 2019-11-09

**Authors:** Fanny Carina Ossa, Reinhard Pietrowsky, Robert Bering, Michael Kaess

**Affiliations:** 1 Department of Clinical Psychology, Institute for Experimental Psychology, Heinrich Heine University Düsseldorf, Universitätsstraße 1, 40225 Düsseldorf, Germany; 2 Center for Psychosocial Medicine, Department of Child and Adolescents Psychiatry, Section for Translational Psychobiology in Child and Adolescent, University Hospital Heidelberg, Blumenstraße 8, 69115 Heidelberg, Germany; 3 Centre of Psychotraumatology, Alexianer-Hospital Krefeld, Dießemer Bruch 81, 47805 Krefeld, Germany; 4 Institute for Clinical Psychology and Psychological Diagnostics, University of Cologne, Klosterstr. 79a, 50931 Cologne, Germany; 5 University Hospital of Child and Adolescent Psychiatry and Psychotherapy, University of Bern, Bolligenstrasse 111, Stöckli, 3000 Bern 60, Switzerland

**Keywords:** Bullying, School victimization, PTSD, Trauma

## Abstract

**Background:**

The aim of this study was to investigate whether bullying among students is associated with symptoms of posttraumatic stress disorder (PTSD), and whether associations are comparable to other traumatic events leading to PTSD.

**Methods:**

Data were collected from 219 German children and adolescents: 150 students from grade six to ten and 69 patients from an outpatient clinic for PTSD as a comparison group. Symptoms of PTSD were assessed using the Children’s Revised Impact of Event Scale (CRIES) and the Posttraumatic Symptom Scale (PTSS-10). A 2 × 5 factorial analysis of variance (ANOVA) with the factors gender (male, female) and group (control, conflict, moderate bullying, severe bullying, traumatized) was used to test for significant differences in reported PTSD symptoms.

**Results:**

Results showed that 69 (46.0%) students from the school sample had experienced bullying, 43 (28.7%) in a moderate and 26 (17.3%) in a severe way. About 50% of the severe bullying group reached the critical cut-off point for suspected PTSD. While the scores for symptoms of PTSD were significantly higher in bullied versus non-bullied students, no significant differences were found between patients from the PTSD clinic and students who experienced severe bullying.

**Conclusions:**

Our findings suggest that bullying at school is highly associated with symptoms of PTSD. Thus, prevention of bullying in school may reduce traumatic experiences and consequent PTSD development.

## Background

Bullying with its negative consequences has become a growing area of interest over the past decade. According to Olweus [1], bullying is defined as negative actions directed against an individual persistently over a period of time where the affected person finds it difficult to defend him/herself against these actions (imbalance of power). In order to prevent stigmatization we call the bully “perpetrator” and the victim “target”. In a large survey of European adolescents, approximately 26% reported to be involved in bullying during the previous 2 months as a perpetrator (10.7%), a target (12.6%), or both a perpetrator and a target (i.e., a bully-victim; 3.6%) [2]. The prevalence varied across countries, age and gender with an overall range of 4.8–45.2% [2].

Bullying by peers is a significant risk factor for somatic and psychological problems, such as psychosomatic symptoms, anxiety and depression, or self-harm and suicidal behavior [3–6]. During young and middle adulthood, previous targets of school bullying are at higher risk for poor general health, lower educational achievement, and having greater difficulty with friendships and partnerships [6]. Studies suggest that school bullying can have long-term effects that are similar to those experienced by targets of child abuse [7]. A recent study reported that children who were bullied only, were more likely to have mental-health problems than children who were maltreated only [8]. Indeed, bullying is a form of aggression, it is intentional and, consistent with the defining features of maltreatment or abuse, can thus be regarded as potential traumatic experience [9]. Some authors have described similarities between the symptomatology associated with being bullied and posttraumatic stress disorder (PTSD), raising the question of whether bullying may lead to PTSD [10, 11].

### PTSD background

The development of PTSD, a mental disorder, can occur in people after they experience or witness a traumatic event, such as a natural disaster, a serious accident, a terrorist act, war/combat, rape, or other violent personal assault. The diagnosis depends on two distinct processes: exposure to a severe trauma (Criterion A) and the development of specific symptom patterns in response to that event (intrusive thoughts, avoiding reminders, negative thoughts and feelings, arousal and reactive symptoms; [12]). Depending on the type of trauma experienced, 10–50% of individuals develop PTSD after experiencing a life-threatening event [13]. A longitudinal study found that 40% of 5 to 18-year-olds experienced at least one traumatic event, and that 14.5% of these children and adolescents and 6.3% of the entire sample had consequently developed PTSD [14]. Although boys are more often subject to traumatic events than girls, some studies report higher rates of PTSD among females [12, 15]. Research shows a higher PTSD prevalence for traumatic events involving interpersonal violence than for natural disasters [16].

To fulfill the diagnostic criteria for PTSD according to the DSM-5, a person must be exposed to a traumatic event (Criterion A), which is defined as direct or indirect exposure to death, threat of death, actual or threat of serious injury, or actual or threat of sexual violence or be a witness of such an event [12]. However, studies have reported even higher symptom rates of PTSD after events actually classified as non-traumatic [17, 18]. Consequently, there is an ongoing debate whether solely Criterion A events are necessary or sufficient to trigger PTSD development [19, 20]. While it is possible that bullying consists of single events with physical violence, which would count as a Criterion A [21], most bullying involves the systematic exposure to non-physical aggression over a prolonged time-period. Thus, most bullying incidents are not officially considered to meeting Criterion A. Nevertheless, bullying meets some of the typical characteristics of a trauma, like its unpredictability or unavoidability. Sometimes affected persons are diagnosed with “adjustment disorder”. This diagnosis is usually applied to individuals who have significant difficulties coping with a psychosocial stressor up to a point where they can no longer sustain their everyday life. Symptoms occur within 3 months of a stressor and last no longer than 6 months after the stressor ends. Stressors that may lead to adjustment disorder can be single events like losing a job or developmental events such as leaving the parents’ home [22]. In the context of bullying this even adds to the injustice done to the targets, as it further accuses them of being incapable of adjusting to the given situation [23]. People should not have to adjust to abuse; they should be protected or defended instead. For bullying targets who, like all other students, spend most of their day at school, it is hard to tell if and when the next attack is imminent. This leads to a permanent state of tension and a feeling of helplessness. Since school is mandatory the daily contact with the abusers cannot be avoided. Targets commonly receive no or just little help or support [24]. For some students, bullying continues into their out-of-school life, e.g. approximately 25% of the bullied students had also experienced cyberbullying in the past [25], and another group suffers from sibling bullying at home [26]. For them there is even less escape, neither at school nor at home.

To fully examine the question if experiences of bullying may trigger the development of PTSD, more studies have to investigate symptoms of posttraumatic stress in bullying targets. A few did so: In an adult sample, Matthiesen and Einarsen [10] found a notably higher symptom level of PTSD among bullying targets in comparison with two groups that had experienced trauma (soldiers from Bosnia and parents who had lost children in accidents). Mynard et al. [27] assessed trauma among school children and found bullying rates of 40% in a sample of 331 adolescents, of which 37% exceeded the symptom cut-off point for PTSD. There were no statistical differences between the prevalence rates of boys (33.9%) and girls (38.7%). In a study by Idsoe et al. [28], the scores of one-third of school bullying targets also reached clinical significance on the study’s traumatic-symptom scales. The chance of falling within the clinical range for PTSD symptoms was about twice as high for girls as for boys. A strong association was found between the frequency of bullying and symptoms of PTSD. In a meta-analysis, Nielsen et al. [11] reported a correlation of .42 (averaged) between school or workplace bullying and symptoms of PTSD. On average, 57% of the targets exceeded the clinical threshold on the traumatic-symptom scales. The authors found that the association between bullying and symptoms of PTSD was equally strong in children or adults.

Approximately one-third of bullied school children show noticeable results on trauma-related questionnaires of PTSD symptoms [27, 28]. However, these data have not been verified by the use of controls with the same environmental conditions (e.g. competition, pressure to achieve, stress caused by exams or application procedures, or experience of other traumatic events), because students without bullying experiences did not have to complete the same questionnaires, nor have they been compared to a traumatized sample in the classical sense. To our knowledge, there are no studies comparing PTSD symptoms in bullied versus traumatized adolescents from a specialized outpatient clinic. In order to judge whether PTSD symptoms of bullying targets are similar to those of traumatized patients, a control group matched by age and gender is necessary. Most of the studies on bullying and its potential for trauma have been conducted with adults. Some of them have investigated participants of anti-bullying programs, a help-seeking clientele, which possibly led to selection bias [10], others were asked to recall their worst school experiences (in retrospect, with a gap of several years between the event and recall), which possibly led to recall bias [29, 30].

The aim of this study was to examine the symptom level of PTSD among targets of bullying at school. We also inquired about how targets’ symptoms related to the duration and frequency of bullying, expecting higher symptom levels of PTSD among those who experienced more frequent bullying. Although previous studies have investigated the correlation between school bullying and posttraumatic stress, they did not make a direct comparison of a bullying sample with a control group in the same environment or with a traumatized group of the same age. Thus, the specific aims of the study were (1) to compare the bullying group to a group of students without bullying experiences, but from the same school with equivalent environmental conditions. We expected that bullying would be associated with higher symptom levels of PTSD in the school sample and (2) to compare the bullying group to a traumatized group matched for gender and age. The aim was to investigate whether bullying targets suffer from similar levels of PTSD symptoms compared to adolescents with other traumatic experiences. Therefore, we expected an equivalent symptom level between students who were severely bullied compared to a group of traumatized children and adolescents who fulfilled Criterion A for PTSD (recruited from a specialized outpatient clinic).

## Methods

### Participants and procedure

The study was conducted in accordance with common ethical standards and was approved by the appropriate institutional review board (Aufsichts- und Dienstleistungsbehoerde, reference number: 51 111-32/20-13). Written informed consent was obtained from the children’s caregivers and subsequently, from the adolescents through their voluntary completion of the questionnaire.

Participants of the school-based sample were recruited from a German secondary public school. In total, 258 students from twelve classes, grades 6, 7, 8, and 10 were asked to participate in the survey. The total response rate was 58.1% and the final sample was *n *= 150 (boys: *n *= 68; mean age = 13.8; range = 11–18 years). The questionnaires (duration 30–45 min) were completed in a classroom under exam-like conditions, and were anonymously returned directly to the researchers.

The clinical sample included 69 patients (boys: *n *= 33; mean age = 13.7; range = 10–18 years) from an outpatient clinic that treated people for PTSD. The clinical sample was matched for gender and age to the total bullying group. After the initial consultation at the outpatient clinic, the patients returned for a second appointment for diagnostic and research assessment including the questionnaires used in this study. At this point, the patients had not yet received any therapeutic help other than the initial consultation. Their reasons for participating in therapy included experiences of sexual abuse (*n *= 20, 29.0%), physical violence/abuse (*n *= 16, 23.2%), death of a family member (*n *= 10, 14.5%), accident (*n *= 4, 5.8%), crime (*n *= 2, 2.9%), escape from war and displacement (*n *= 2, 2.9%), critical illness (*n *= 1, 1.4%), and other events (*n *= 14, 20.3%; e.g., witness to severe violence or house break-in; threat of murder). The questionnaires were part of the diagnostic process prior to a clinical interview. Among the clinical-sample, 52 (75.4%) were diagnosed with PTSD (F43.1) according to the ICD-10 diagnostic criteria [31], 12 (17.4%) were diagnosed with “other reactions to severe stress” (F43.8) and 5 (7.2%) with “adjustment disorder” (F43.2). Thirty-seven (53.6%) patients suffered from comorbid depression and 8 (11.6%) from anxiety disorder.

### Measures

Bullying was measured using a questionnaire specifically designed to suit the study. The students were first given a written explanation of bullying behavior, according to Olweus [32], followed by questions such as (1) “Have you ever been bullied?” with the response categories “yes” and “no”; “How long has the bullying been going on (currently or in the past)?”, with the possible answers categories: “I’m not being bullied”, “I have been bullied between grade __ and grade __”; “more than 2 years”; “more than 1 year”; “more than 6 months”; “less than 6 months”; “more than 2 months”; “less than 2 months”. (2) “How often are you being/have you been bullied?” with the categories “I’m not being bullied”; “several times a day”; “once per day”; “almost every day”; “once per week”; “once per month”; “once in 3 months”; “infrequent”. (3) “If you are/were a target of bullying, how long ago has that been?” with the categories: “I’m still being bullied”; “it is 2–4 weeks ago”; “it is more than 4 weeks ago”; “it is more than 2 months ago”; “it is more than 6 months ago”; “it is more than 1 year ago”; “it is more than 2 years ago”. In the literature, a current target is usually defined by at least “two or three times per month” during the last 3 month. For more serious cases, Solberg and Olweus [5] set a cut-off point for the frequency of weekly incidents and Leymann [33] reported notably worse consequences after exposure to bullying for at least 6 months. Therefore, the study at hand differentiated moderate (less than 6 months and/or less than once per week) from severe bullying (at least 6 months and once per week).

Additional two questions with examples for physical and verbal aggression were provided. The questions were “Did one of these things happen to you in the past?” followed by a list of possible examples like “I was physically threatened”; “I was laughed at”; “I was insulted”; “Classmates made fun of me” and the option to select several answers. None of the actions described bullying per se. If verbal or physical aggression happens occasionally or between two parties with similar power, this refers to aggressive or conflict behavior at school but not to bullying. In order to control how conflicts (same actions but no bullying) affect mental health, all students completed these questions (not just the targets of bullying). If students selected one or more of these items and responded at the same time that they had not been bullied in the past, they were counted among the conflict group. The purpose of these questions was to explain the bullying situation more specifically (for the bullying groups) and differentiate a conflict group from those who were bullied.

Symptoms of posttraumatic stress were measured using the Children’s Revised Impact of Event Scale (CRIES; [34]) and the Posttraumatic Symptom Scale (PTSS-10; [35]). The CRIES is a 13-item scale assessing three dimensions of symptoms often reported after a traumatic event: avoidance, intrusion, and arousal. The total score includes the two subscales intrusion and avoidance. A cut-off point of 17 maximizes the instrument’s sensitivity and specificity, thereby minimizing the rate of false negatives and classifying 75–83% of children correctly [36]. In the present study, Cronbach’s alpha for the overall scale was .91. Patients from the clinical sample who were older than 14 years completed the adult version of the CRIES, referred to as the IES-R [37]. Yule (1997, cited by [36]) found a correlation of *r *= .95 between both versions. Therefore, for every question on the CRIES, the corresponding question on the IES-R was used in the statistical analysis. The PTSS-10 contains ten problems that indicate the presence of PTSD: (1) sleep problems, (2) nightmares about the trauma, (3) depression, (4) startle reactions, (5) tendency to isolate oneself from others, (6) irritability, (7) emotional lability, (8) guilt/self-blame, (9) fear of places or situations resembling the traumatic event, and (10) muscular tension. A score of 24 or higher indicates PTSD (Weisæth and Schüffel, personal communication cited by [38]). Cronbach’s alpha was found to be .92 in the present study. The correlation between CRIES and PTSS-10 scores was *r *= .80 (*p *< .01, *N *= 214). The CRIES asks for situations which are directly related to the stressful event (e.g. “Do you try not to think *about it*?” or “Do pictures *about it* pop into your mind?”). The PTSS-10 asks for symptoms such as sleep problems or muscular tension, which could also be triggered by other stressful events (exam stress, stress at home). Both scales assess characteristic symptoms of PTSD, which is why both instruments were used in this study.

In contrast to previous research, both bullied and non-bullied students were asked the symptom scales, resulting from bullying or from other threatening life events. If non-bullied students had experienced a threatening life event, they were instructed to respond to the CRIES questions in relation to this specific situation. If not, the adolescents were asked to assign a rating of zero to the relevant questions (e.g., “Do pictures *about it* pop into your mind?”). The bullying group was instructed to relate their bullying situations to their responses to the CRIES questions. However, they were allowed to indicate whether they had experienced any additional serious life events. The request to describe the serious life event in more detail was optional. In the analysis of the results, we examined this sample separately. We performed two calculations: the first one included the entire sample and the second excluded all children who reported at least one additional serious life event, to avoid bias due to additional serious life events.

### Data analysis

Data analyses were conducted with SPSS [39]. A 2 × 5 factorial analysis of variance (ANOVA) with the factors of gender (male, female) and group (control, conflict, moderate bullying, severe bullying, traumatized) was used to test for significant differences in reported symptoms. Scheffé’s post hoc tests were used. Chi square tests were used to compare non-parametric data. To proof the statistical dependence between parametric data we used the Pearson correlation coefficient. For non-parametric data we used Spearman’s rank correlation coefficient. The alpha level for all analyses was < .05. Of the 219 participants included in the study, 7 (3.2%) were missing one or more items in the trauma related questionnaires. N = 1 participant had one and n = 1 participant had two missing items in the PTSS-10. The data from both participants were included in the analyses and the missing items were counted as zero. N = 2 participants had more than two missing items in the CRIES and n = 5 participants had more than two missing items in the PTSS-10. The results from theses participants (n = 7) were excluded from the data analyses. Missing items were found in every group inside the school sample.

## Results

Of the study’s 150 students, 69 (46.0%) reported victimization by bullying in the past. In each of the 12 classes, between 2 and 11 targets were found. The school sample was grouped as follows: (1) control (no bullying and no conflicts in the past), (2) conflict (some trouble or conflicts with others, but would not call this bullying), (3) moderate (less than 6 months and/or less than once per week), and (4) severe bullying (at least 6 months and once per week) (see Table 1). A Chi square test showed that boys and girls were equally likely to be in either group (χ_(2)_^2^ = .81, *p *= .667). Each group consisted of students who reported additional serious life events (see Table 1).

**Table 1:** Frequency distribution of the groups (total sample) and number of students per group, who reported a serious life event other than bullying

	School-sample										Clinical-sample	
	Control		Conflict		Moderate bullying		Severe bullying		Total		Traumatized	
	N	%	N	%	N	%	N	%	N	%	N	%
Total	45	30.0	36	24.0	43	28.7	26	17.3	150	100	69	100
Girls	24	53.3	22	61.1	24	55.8	12	46.3	82	54.6	36	52.2
Boys	21	46.7	14	38.9	19	44.2	14	53.8	68	45.3	33	47.8
Additional serious life event	10	22.2	10	27.8	8	18.6	4	15.4	32	21.3	–	–

In the overall bullying group, 37.1% of the girls and 65.6% of the boys reported at least one physical attack; 97.1% of the girls and 96.9% of the boys reported verbal bullying; 73.9% experienced bullying at school, 21.7% via the internet, 4.3% via mobile phone, and 8.7% reported other places (on their way to school, outside). 20.3% students chose more than one answer. Among 55.9% of the students in the overall bullying group, the bullying occurred during the previous year and 8.7% of the bullying group (4% of the total sample) fulfilled the criteria for severe bullying at the time the sample was taken.

### Children’s Revised Impact of Event Scale (CRIES)

**Fig. 1:**
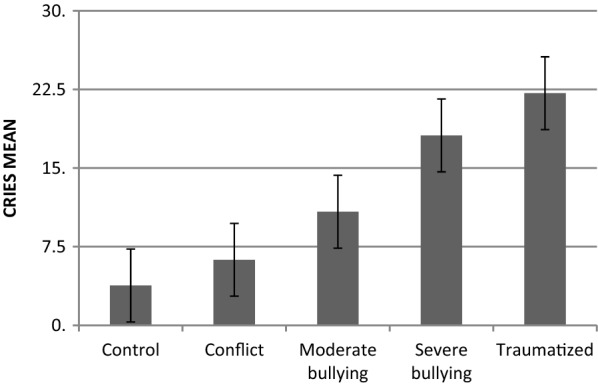
This graph displays the means of the CRIES score (intrusion and arousal) for each group. The error bars indicate the standard error

The ANOVA of the total sample (*N *= 217) showed a significant main effect of group (*F*_(4/207)_ = 35.67, *p *< .001, *η*^*2*^ = .41). There was no significant main effect of gender (*F*_(1/207)_ = 3.00, *p *= .085, *η*^*2*^ = .01) and no significant interaction between group and gender (*F*_(4/207)_ = .58, *p *= .681, *η*^*2*^ = .01). Means, standard deviations, ranges, and group sizes are presented in Table 2. The exclusion of students with additional life events had no effect on the main results (values in brackets in Table 2). The mean scores on the CRIES for each group are displayed in Fig. 1.

**Table 2:** Means, standard deviation, minimum and maximum values from the CRIES combined score (intrusion and avoidance) and PTSS-10 measuring traumatization symptoms

	CRIES								PTSS-10							
	All traumatic events				Bullying victimization only				All traumatic events				Bullying victimization only			
	M	SD	Min–max	N	M	SD	Min–max	N	M	SD	min–max	N	M	SD	Min–max	N
School																
Total	8.91	9.59	0–34	148	6.65	8.50	0–32	116	11.20	12.27	0–55	145	8.15	10.21	0–55	114
Control	3.80	7.05	0–25	44	0.91	2.43	0–10	34	6.28	8.45	0–34	43	3.33	4.73	0–20	33
Conflict	6.25	7.91	0–30	36	2.65	4.92	0–20	26	8.34	10.48	0–35	35	4.96	7.28	0–33	26
Moderate bullying	10.83	8.89	0–34	42	8.82	7.27	0–25	34	13.02	10.53	0–39	41	9.03	6.65	0–24	33
Severe bullying	18.12	9.34	0–32	26	16.86	9.20	0–32	22	20.31	16.62	0–55	26	17.82	15.75	0–55	22
Clinic																
Traumatized	22.14	10.86	0–40	69	–	–	–	–	28.67	14.04	4–56	69	–	–	–	–

The table displays the values of the total school sample with all kinds of traumatic events and the subsample after excluding students with additional serious life events other than bullying (bullying victimization only)

The Scheffé post hoc tests revealed that there was no significant difference between the severe bullying group (*M *= 18.12, *SD *= 9.34) and the traumatized group (*M *= 22.14, *SD *= 10.86, *p *= .451). Results are shown in Table 3. Even after excluding of students with additional serious life events no statistical difference was found between the severe bullying group (*M *= 16.86, *SD *= 9.20) and the traumatized group (*p *= .147).

**Table 3:** p-values from Scheffé post hoc tests for the CRIES score (intrusion and avoidance) and the respective effect size Cohen’s d

	Conflict	Moderate bullying	Severe bullying	Traumatized
Control				
p-value	0.838	0.014	<0 .001	< 0.001
Cohen’s d	0.3	.9	1.7	2.0
Conflict				
p-value	–	0.301	< 0.001	<0 .001
Cohen’s d	–	0.5	1.4	1.7
Moderate bullying				
p-value		–	0.040	< .001
Cohen’s d		–	0.8	1.1
Severe bullying				
p-value			–	0.451
Cohen’s d			–	0.4

*N * = 50 (72.5%) students in the traumatized group, *n * = 16 (61.5%) in the severe bullying group, *n * = 10 (23.8%) in the moderate bullying group, *n * = 5 (13.9%) in the conflict group, and *n * = 4 (9.1%) in the control group had scores within the clinical range (≥ 17 points). Group differences were significant (χ^2^_(4)_ = 68.08; *p *< .001). No difference was found between the traumatized and the severe bullying group (χ^2^_(1)_ = 1.06; *p * = .303). Boys and girls were equally likely to score within the clinical range (χ^2^_(1)_ = .60; *p * = .438). After exclusion of those who reported an additional serious life event, *n *= 13 (59.1%) in the severe bullying group, *n * = 5 (14.7%) in the moderate bullying group, *n * = 1 (3.8%) in the conflict group, and 0 in the control group had scores within the clinical range. Group differences were significant (χ^2^_(4)_ = 81.04; *p * < .001). No difference between the traumatized and the severe bullying group was found (χ^2^_(1)_ = 1.40; *p * = .237).

We correlated CRIES scores with duration, frequency and elapsed time for the overall bullying group. A significant relationship (Spearman`s correlation, one tailed) between duration (*r*_*s*_ = .29, *p * = .009) and CRIES scores as well as frequency of bullying (*r*_*s*_ = .39, *p * < .001) and CRIES scores was found. The elapsed time since the last bullying incident had no significant influence on the CRIES scores (*r*_*s*_ = − 0.15, *p * = .118). Within the traumatized group, no significant interrelationship between the elapsed time since the occurrence of the traumatic event and CRIES scores was found (*r*_*s*_ = .11, *p * = .176).

### Posttraumatic Symptom Scale (PTSS-10)

**Fig. 2:**
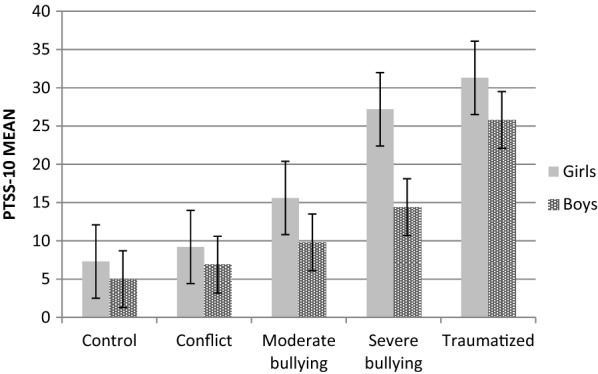
This graph displays the means of the PTSS-10 score for each group and gender. The error bars indicate the standard error

The 2 × 5 factorial ANOVA conducted with the total sample (*N * = 214) showed a significant main effect of group (*F*_(4/204)_ = 31.01, *p * < .001, *η*^*2*^ = .38) and gender (*F*_(1/204)_ = 10.71, *p * = .001, *η*^*2*^ = .05). The interaction between group and gender was not significant (*F*_(4/204)_ = .92, *p * = .453, *η*^*2*^ = .02). Means, standard deviations, ranges, and group sizes are reported in Table 2. The exclusion of students with additional serious life events had no effect on the main results (values in brackets Table 2). The means of the PTSS-10 scores for each group separated by gender, including those who reported additional serious life events, are displayed in Fig. 2.

The Scheffé post hoc tests revealed that there was no significant difference between the severe bullying group (*M * = 20.31, *SD * = 16.62) and the traumatized group (*M *= 28.67, *SD * = 14.04, *p * = .062). The results after the post hoc analysis (Scheffé) and the effect sizes (Cohen’s d) are shown in Table 4. After excluding those who had reported an additional serious life event, no significant differences between the severe bullying (*M * = 24.00, *SD * = 16.94) and traumatized groups (*M * = 31.31, *SD * = 14.11) were found for the girls (*p * = .520). The difference between the severe bullying and the traumatized group was significant for the boys and for the total score (*p *< .05).

**Table 4:** p-values from Scheffé post hoc tests for the PTSS-10 and the respective effect size Cohen’s d

	Conflict	Moderate bullying	Severe bullying	Traumatized
Control				
p-value	0.966	0.162	<0 .001	<0 .001
Cohen’s d	0.2	0.7	1.1	1.9
Conflict				
p-value	–	0.582	0.006	<0 .001
Cohen’s d	–	0.5	0.9	1.7
Moderate bullying				
p-value		–	0.216	<0 .001
Cohen’s d		–	0.5	1.3
Severe bullying				
p-value			–	.062
Cohen’s d			–	0.5

A total of *n *= 45 (65.2%) students in the traumatized group, *n *= 12 (46.2%) in the severe bullying group, *n *= 8 (19.5%) in the moderate bullying group, *n *= 5 (14.3%) in the conflict group, and *n *= 2 (4.7%) in the control group had scores within the clinical range (≥ 24 points). The group differences were significant (χ^2^_(4)_ = 58.40; *p *< .001). No group differences were found between the traumatized and the severe bullying groups (χ^2^_(1)_ = 2.86; *p * = .090). Girls and boys were equally likely to score within the clinical range (χ^2^_(1)_ = 2.68; *p * = .100). After excluding those who had reported an additional life event, *n * = 9 (40.9%) in the severe bullying group, *n * = 1 (3.0%) in the moderate bullying group, *n *= 1 (3.8%) in the conflict group, and 0 in the control group scored within the clinical range. Group differences were significant (χ^2^_(4)_ = 75.16; *p *< .001). The difference between the traumatized and severe bullying group was now significant with higher scores for the traumatized group (χ^2^_(1)_ = 4.09; *p *< .05).

Among the students in the overall bullying group, no significant relationships (Spearman`s correlation, one tailed) between the total score in the PTSS-10 and duration (*r*_*s*_ = .20, *p *= .057), frequency (*r*_*s*_ = .14, *p *= .134) and the elapsed time since the last bullying incident (*r*_*s*_ = − .05, p = .340) were found. Among students in the traumatized group, no significant interrelationship was found between the elapsed time since the occurrence of the traumatic event and the PTSS-10 scores (*r*_*s*_= − .02, *p *= .435).

## Discussion

Bullying is a universal social-health problem, having an impact on a large number of adolescents. In our study, 46% of the school sample reported involvement in bullying as current or former targets. Earlier studies have found similar prevalence rates ranging from 40 to 43% [27, 28]. An additional 24% of the students had prior involvement in school conflicts or victimization. Although the definition criteria for bullying were not fulfilled by the conflict group, the study showed a high prevalence of school victimization in a representative sample of school children in Germany (70%). In accordance with the discussion of earlier research [5, 33], and the recommendation of Fischer and Riedesser [38], that the term bullying in the context of psychological traumatology should be reserved to describe a “severe, potentially traumatic situation”, we differentiated moderate from severe bullying. Our results showed that 40% of the overall bullying group comprised the severe bullying group, which was comparable to the findings of Solberg and Olweus [5], who reported that among targets of bullying 38.3% were bullied at least weekly in the last couple of months. Altogether, every sixth student (17.3%) was subject to severe bullying according to our definition (longer than 6 months and more than once per week). This finding supports Rigby [40] who reported that 15% of the school sample had been bullied once a week or more. Although the association between the frequency or duration of bullying and symptoms of PTSD were examined in earlier research, as far as we know, the combination of duration and frequency has rarely, if ever, been investigated before. In line with Mynard et al. [27], boys and girls were equally likely to have been bullied. However, these results are in conflict with other studies that report more targets among boys [5, 28].

### Bullying and posttraumatic stress

Results show a high symptom level of PTSD among bullied students. Around 50% (range 46.2–61.5%) of the severe bullied adolescents had scores within the clinical range. These findings are consistent with the meta-analysis by Nielsen et al. [11] in which, on average, 57% of bullied persons reached the clinical threshold in PTSD questionnaires. In our clinical sample for comparison, 65.2%–72.5% reached the critical range with no significant differences between the severe bullying group and the clinical sample. This suggests that severe bullying targets show clinically relevant symptoms of PTSD. Matthiesen and Einarson [10] compared adult targets of bullying to a traumatized group using the PTSS-10, and reported even higher symptom rates among the bullying targets. This result might be explained by the type of recruitment because their bullying group was recruited from a help seeking population. In our study, the traumatized sample was drawn from a help-seeking population, whereas the severely bullied students were recruited from a randomly selected school sample.

Maltreated children are more likely to be bullied than children who have not been maltreated [8]. Therefore, high scores on PTSD symptom questionnaires could potentially be caused by experiences of serious and adverse life events in the past. To alleviate this potential bias in our analysis, we excluded this group from a second sensitivity analyses. Although the statistical effects were slightly reduced, the severe bullying and clinical groups reached parity on the PTSD symptom scales even after the exclusion of those with additional experiences (CRIES). Additionally, the PTSS-10 scores were still high among those in the severe bullying group, especially girls. Furthermore, the severe bullying group still showed the greatest risk of reaching critical scores (40.9–59.1%, controls = 0%). As the exclusion of students with additional serious life events did not change our main results, it is likely that the high scores are specifically associated with bullying and not largely influenced by multiple traumatic events. This finding confirmed our hypothesis that symptoms of PTSD mainly resulted from bullying, supporting Nielsen et al. [11], who found that PTSD symptoms were overrepresented in bullying targets. Thus, prevention of bullying at school may reduce traumatic experiences and consequent PTSD development.

In the PTSS-10 girls scored higher than boys. This is consistent with studies reporting higher rates of PTSD among females within the general trauma field [12, 15]. Questions remain on whether gender is a risk factor for PTSD per se or if this effect is influenced by characteristics such as levels of symptom reporting, e.g. women have been shown to be more willing to disclose traumatic experiences than men [15]. However no gender differences could be found in the CRIES where boys and girls were equally likely to score within the clinical range. The inconsistent gender effect within our study may point to the methodological problem of heterogeneity in definitions and operationalization of PTSD symptom measures [41]. Interestingly, our CRIES results are similar to Mynard et al. [27] who found no gender differences in the long version of the CRIES (Impact of Events Scale; [37]) but contrary to Idsoe et al. [28] who found higher rates for girls in the CRIES and more girls who reached the clinical range. Overall, gender differences in PTSD symptoms might arise due to questions that are more applicable or even just easier reportable for girls (like nightmares and anxiety) while boys tend to deny these symptoms because of their social role. As another hypothesis, girls tend to cope with stressors by asking for social support [42]. If this support is affected by bullying and exclusion it may be more difficult for girls than for boys to solve their problems on their own, resulting in higher levels of PTSD symptoms [41]. Overall, the results on gender differences of PTSD symptoms remain inconsistent (in particularly with regards to bullying and PTSD symptoms); therefore further studies should examine gender specific reactions and coping strategies following bullying among adolescents.

As expected, there was a linear trend in the degree of PTSD symptoms and experiences of verbal or physical aggression (control group < conflict group < moderate bullying group < severe bullying group). The conflict group showed slightly more symptoms than the control group, but fewer symptoms than the moderate bullying group. Given the definition of bullying stating that targets of bullying are unable to defend themselves [32], one might assume that the conflict group represents harassed students who can defend themselves rather than become helpless [43]. Contrary to the discussion that the use of the term bullying is inflated [44], we found a group of students who experienced peer aggression but did not assign the term carelessly; they were able to discern between bullying and other kinds of victimization. Further research should reveal whether this group is more likely to become bullying targets in the future, or if they might be even more resilient.

In the CRIES, the severe bullying group reached clinical ranges of scores indicating higher levels of PTSD symptoms, i.e. three times more often (61.5%) than the moderate bullying group (23.8%). The interrelationship between the symptoms in the CRIES and duration and frequency of bullying is also reflected in the significant correlation scores. Hence, duration and frequency of bullying had a considerable influence on the level of symptoms in the CRIES. In the PTSS-10, twice as many students of the severe as the moderate bullying group reached the clinical range (46.2% vs. 19.5%). The differences in the averages between the severe and the moderate bullying group, however, was not significant, which is also reflected in the non-significant correlations of duration and frequency with the PTSS-10 scores. Hence, longer or more frequent bullying did not lead to more symptoms in the PTSS-10. Although further research is necessary, these results might suggest that there is a critical threshold where longer duration and higher frequency is no longer associated with an increased severity of PTSD symptoms.

The elapsed time since the events did not automatically lead to a decrease in the symptoms, neither in the traumatized, nor in the bullying groups. This underscores the relative time stability found in other research, which characterizes PTSD [10, 12] contrary to adjustment disorder where the symptoms last no longer than 6 months [22]. This implies that bullying in children and adolescents may negatively affect their wellbeing, even months or years after an incident. Other studies also note the long-term effects of bullying [6]. Furthermore, this gives weight to the assumption that the students’ symptoms are more than simple stress reactions or short bursts of mood swings in response to negative experiences, indicating that this group of students is a clientele that needs help. In the present study, the presence of symptoms, even after the bullying had ceased, can also be explained in part, by external factors. As schooling is mandatory, students are reminded regularly of their negative experiences by the setting and ongoing contact with their abusers. Our study and the literature show that bullying is associated with the three symptom clusters of PTSD [11]. A discussion on whether or not bullying constitutes a causal factor of PTSD development is indicated. If so, the current validity of the Criterion A needs reviewing. Other authors have already questioned the functionality of PTSD diagnostic criteria [18, 20]. Van Hoof et al. claim that the clarification of events as either traumatic or non-traumatic is determined by rater’s subjective interpretation of the diagnostic criteria, and hence a matter of opinion [18]. At the moment, bullying targets receive little or no help to deal with their short and long-term consequences. A proper diagnosis could increase support and treatment availability to those affected. This is even more important as post-event factors may play a major role in determining whether or not a child develops PTSD following a traumatic event [45]. Further research should investigate whether access to PTSD treatments could support bullying targets to cope with long-term effects.

### Limitations

A limitation of the study is that it did not assess all students because written informed consent could only be obtained from 58.1% of their caregivers. A higher rate would have been desirable to increase the representativeness of the sample. Students affected by intense bullying at the time might have objected to participation in the survey because of avoidance. As bullying often leads to school absenteeism [44, 46], this factor should be considered when interpreting the data. In addition, assessments of bullying using self-report questionnaires have been criticized for their subjectivity. A more precise depiction of both perpetrators and targets could be obtained through additional reports from parents, teachers, and peers. Measuring symptoms of PTSD with a questionnaire cannot substitute a full diagnostic. A follow-up screening including a clinical evaluation would be useful to see whether bullied students do not only display symptoms of PTSD, but can actually be diagnosed with PTSD. Although we tried to control for previous traumatic life-events within our sensitivity analyses, the study did not address premorbid psychiatric history or pathological personality traits that could potentially influence both the development of bullying and PTSD. In addition, bullying was not assessed within the clinical sample, which should be done in future research. Another factor is the limiting generalizability of our results for all subgroups due to their small sample size. Replication studies with lager case numbers, especially for the severe bullying group, would be fairly recommended. Finally, it should be noted that conclusions on the direction of the relationship between bullying and symptoms of PTSD cannot be drawn from our study, although we expected the occurrence of PTSD symptoms as a consequence of bullying.

## Conclusion

This study once more demonstrated the high burden of bullying on mental health. Targets of severe bullying had similar symptom patterns (intrusion/avoidance/arousal) compared to adolescents seeking help at an outpatient clinic for PTSD. Our results suggest that bullying may be regarded as one type of traumatic experience that can potentially cause PTSD. Thus the results indicate that bullying prevention in schools may reduce traumatic experiences and consequent PTSD symptom development. A large proportion of students reported bullying experiences within school, and many of them reported relevant symptoms of PTSD even after the bullying ceased. In terms of everyday school life, this means that these adolescents suffered from symptoms, such as concentration difficulties, nightmares, sleep disorders, depression, and fear of intrusive thoughts and feelings, which likely has implications for the quality of both education and life. Thus, bullying prevention should become a major focus for both educational and public health authorities. However, not only bullying prevention is implicated. Our results show that children may suffer from PTSD symptoms long after a cessation of bullying episodes. Thus, early intervention is warranted for targets of bullying, and evidence-based treatments that are available for trauma-related disorders could be adapted to and implemented within the school context [9].

## Data Availability

The datasets used and/or analyzed during the current study are available from the corresponding author on reasonable request.
